# Factors Contributing to Hypoxia in the Minjiang River Estuary, Southeast China

**DOI:** 10.3390/ijerph120809357

**Published:** 2015-08-11

**Authors:** Peng Zhang, Yong Pang, Hongche Pan, Chengchun Shi, Yawen Huang, Jianjian Wang

**Affiliations:** 1 College of Environment, Hohai University, 1 Xikang Road, Nanjing 210098, China; E-Mails: zhap2014@163.com (P.Z.); panhongche@126.com (H.P.); hywbby@163.com (Y.H.); hhuwangjianjian@126.com (J.W.); 2 Key Laboratory of Integrated Regulation and Resource, Development on Shallow Lakes of Ministry of Education, Hohai University, Nanjing 210098, China; 3 Fuzhou Research Academy of Environmental Sciences, 32 Jinjishan Road, Fuzhou 350013, China; E-Mail: stonerainman@126.com

**Keywords:** hypoxia, dissolved oxygen, oxygen depletion, water resources security, Minjiang River Estuary

## Abstract

Dissolved oxygen (DO) is not only a fundamental parameter of coastal water quality, but also an indication of organics decomposed in water and their degree of eutrophication. There has been a concern about the deterioration of dissolved oxygen conditions in the Minjiang River Estuary, the longest river in Fujian Province, Southeast China. In this study, the syntheses effects on DO was analyzed by using a four year time series of DO concentration and ancillary parameters (river discharge, water level, and temperature) from the Fuzhou Research Academy of Environmental Sciences, at three automated stations along the Minjiang River Estuary. Hypoxia occurred exclusively in the fluvial sections of the estuary during the high temperature and low river discharge period and was remarkably more serious in the river reach near the large urban area of Fuzhou. Enhancement of respiration by temperature and discharge of domestic sewage and industrial wastewater, versus regeneration of waters and dilution of pollutant concentration with increased river discharge, which regarded as the dominant antagonist processes that controlled the appearance of seasonal hypoxia. During the high temperature and the drought period, minimal mainstream flow above 700 m^3^·s^−1^, reduction of pollutants and forbidding sediment dredging in the South Channel should be guaranteed for strong supports on water quality management and drinking water source protection.

## 1. Introduction

Hypoxia is a condition that occurs in the water column when dissolved oxygen (DO) falls below the 2 mg·L^−1^ (or 62.5 μmol·L^−1^) level necessary to sustain most animal life [1,2]. Hypoxia results in deaths of phytoplankton and zooplankton, anaerobic decomposition of the subsequent settling of organic matters, deterioration of water quality and variation and loss of the habitats [3,4]. The security of aquatic ecosystems and water source areas close relating to public health is under serious threat in hypoxic waters. A lot of factors contribute to the temporal variability and spatial distribution of DO, such as freshwater and groundwater inputs, water volume and tidal advection and dispersion in estuary which will determine the residence time of waters and their resistance to increasing nutrient fluxes and eutrophication [5]. In addition, high temperature, excess nitrification, sediment oxygen demand (SOD) and transformation of nutrient and organic matter loading from drainage basins *et al.* may also determine estuary susceptibility to hypoxia [6,7,8,9]. Better understanding of these factors will help to predict the evolution of oxygen content of water source areas and to elaborate mitigation strategies. More information is needed to determine the full spatial and temporal extent of hypoxic waters and whether their occurrence is increasing or decreasing.

Estuaries, as heterotrophic ecosystems where large numbers of organic substance transported by rivers and inputted from the ocean are mineralized [10,11], have appeared hypoxia and low DO around the world [2,5,12,13,14,15,16,17,18]. For example, a large area of bottom oxygen depletion has been documented in the Yangtze River plume [19,20] and the Pearl River Estuary [1,17] respectively. An alarming “dead zone” with DO below 2 mg·L^−1^ has been found in the northern Gulf of Mexico which is the largest zone of oxygen-depleted coastal waters in the United States [14,21]. European estuaries, such as Gironde, Scheldt, Seine, Forth, are also oxygen depleted to different degrees, largely attributable to anthropogenic factors [5,22,23,24,25].

However, there is an increase of peer-reviewed articles reporting the occurrence of seasonal hypoxia in bottom waters in either salt wedge estuaries or on coastal shelves during stratifying seasons (typically in late spring and summer), mainly ascribed to eutrophication-induced high biological productivity and restricted water exchange [2,9,12,15,26,27]. In this paper, a case of hypoxia expanded in the surface water of water source areas in the Minjiang River Estuary located at the city of Fuzhou was reported. Special effort is given here to identify the synthesis of different factors influencing oxygen consumption, including freshwater, tidal, high temperature, and transformation of nutrient and organic matter loading from drainage basins *et al.* The daily average hydrological and water quality data from the three automated continuous monitoring stations were analyzed to explore the seasonal patterns and correlation between DO and river discharge/temperature. The evolution of water quality along the Minjiang River, as well as the probable trends in the occurrence of hypoxia in the future, was revealed based on the water quality data of seven routine monitoring points from 1995–2013.

## 2. Research Area

**Figure 1:**
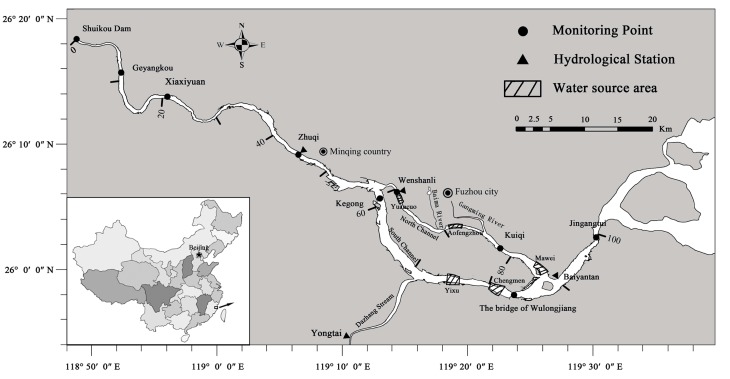
Location of the study area: the Minjiang fluvial-estuarine system and its hydrological stations, monitoring points and water source areas. Numbers indicate the distance in km from Shuikou Dam.

The Minjiang River is the longest river (2959 km) in Fujian Province, Southeast China. The Minjiang River Estuary is located close to Fuzhou City (Figure 1), which is the capital of Fujian Province [28]. On the Minjiang watershed (59,922 km^2^ in Fujian Province), rainfall is the highest in summer and the smallest in autumn. The annual average discharge of the Minjiang River is 1760 m^3^·s^−1^, which is the seventh highest annual runoff in China [29]. The discharge varies seasonally, reaching a maximum in April–July (average 3200 m^3^·s^−1^) [30] and a minimum in October–March (average 620 m^3^·s^−1^). The annual average water temperature is 19.9 °C with a range of 9.8–32.2 °C. Minjiang River Estuary is divided into two branches (the South Channel and the North Channel) in upstream of the Wenshanli, and then merged at the Baiyantan. Minjiang River is approximately 200 m at the Shuikou Dam and gradually broadens to about 2 km at the Baiyantan. The mean depth of the river is −3 m in the upstream with a maximum depth of −30 m near the downstream. The average width of the South Channel is 1 km, wider than the North Channel with the width of 0.5 km. However, the average bottom elevation of the North Channel is 2–3 m deeper than that of the South Channel.

In recent years, the Minjiang River Estuary has a high load of anthropogenic nutrients from increased agricultural activities and wastewater due to the population increase and economic development in Fuzhou City [28]. Several episodes of low dissolved oxygen content have been observed in the river section close to the municipal district of Fuzhou in the high temperature season, in particular during droughts of 2009 and 2011. The water in the North Channel is the most serious areas with oxygen consumption, which leads to deterioration of water quality of Yuancuo, Aofengzhou and Mawei water source areas. There are two reasons: (1) the data show that the river discharge flowing into the north Channel has been significantly reduced due to riverbed entrenchment of the South Channel in recent years [31]; (2) the North Channel receives 80% of domestic sewage and 90% of industrial wastewater of the municipal district of Fuzhou (2,984,900 inhabitants) [32]. Especially, Baima River and Guangming River, as two passages of drainage into the North Channel, is accompanied by water qualities (COD > 40 mg·L^−1^, ammonium > 6 mg·L^−1^) which are seriously substandard.

## 3. Materials and Methods

Zhuqi, Wenshanli and Baiyantan, are three hydrological stations in the Minjiang River, monitoring the daily flow data and tidal level (maximum and minimum values for each tidal cycle) [33]. Since 2010, water quality automonitor has been set in three hydrological stations by Fuzhou Research Academy of Environmental Sciences (FRAES) in the Minjiang River (Figure 1), which provide real-time and continuous measurement of DO, temperature and water quality. Parts of the data has been displayed to the public [34]. Three stations along the Minjiang River Estuary represent the dissimilar estuarine reaches. Zhuqi station, located 45.4 km from Shuikou Dam in upstream, remains influenced by the tide all year round. The other two stations are situated in the tidal reach, respectively at 61 and 90 km from the Shuikou Dam. Wenshanli, located in upstream of the North Channel, is in the municipal district of Fuzhou suburb of about 2,984,900 inhabitants. The automated stations are all based on the same architecture, including an autonomous measurement system and a bidirectional telecommunication system. Water is pumped 1 m below the surface and circulated through a measuring cell including a dissolved oxygen optode (0–20 mg·L^−1^ ± 0.2 mg·L^−1^), a temperature probe (0–35 °C ± 0.1 °C), a pH probe (0–14 ± 0.1). All sensors are adapted to riverine and coastal conditions, and are able to work over long periods without drifts in measured values.

In addition, DO and water quality of seven routine monitoring points (Shuikou, Geyangkou, Xiaxiyuan, Zhuqi, Kuiqi, The bridge of Wulongjiang and Jingangtui in Figure 1) along the Minjiang River were monitored every month by FRAES from 1995–2013. Water samples were collected with Niskin bottles at 1 m below the surface. Dissolved oxygen and temperature were determined using a HQ30d or a YSI ProODO in the field. The concentration of COD, NO_2_^−^, NO_3_^−^, NH_4_^+^, TN and TP were measured using an automatic water quality analyzer (AA3, SEAL, Ludwigshafen, Germany). Before measurement, the water samples were filtered using a water-circulation multifunction vacuum pump (SHB-III, Shiding, Zhenzhou, China).

In order to interpret the factors that control the variations of DO, we performed statistical analysis on daily-averaged data. We compared values of temperature, flow and DO according to stations (Zhuqi, Wenshanli, Baiyantan), period (months and seasons) and their interactions by performing partial correlation analyses. We performed multiple correlation tests and partial correlation analyses (with station as a fixed factor) on daily-averaged data for the four year dataset in order to investigate how temperature and river discharge influence DO.

## 4. Results and Discussion

### 4.1. Spatiotemporal Variations

#### 4.1.1. Variations of the Split Ratio of the North Channel

**Figure 2:**
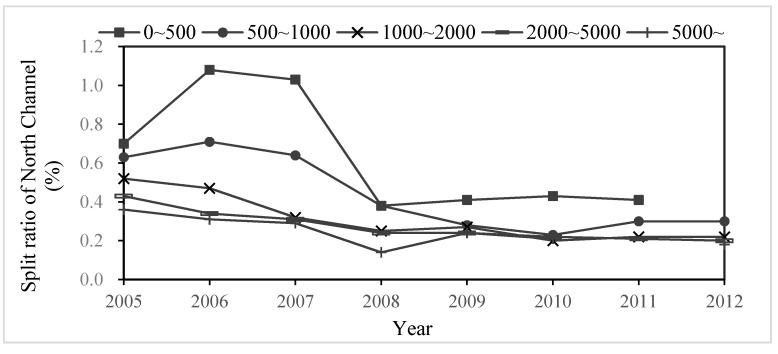
Change of split ratio of North Channel (%) under different discharge grades from 2005–2012.

The split ratio of the North Channel (SRNC) is a proportion of discharge of the North Channel in the total river discharge. Historical data showed that SRNC, affected by the morphology of the North Channel and the South Channel, was 70% during the drought period and 30% during the flood period before 1990. But SRNC had a big change in recent years. We counted SRNC by daily discharge under different grades of discharge of Zhuqi Station from 2005–2012 (Figure 2). The results showed that SRNC had a negative correlation with river discharge at the same hydrological year, for example, SRNC decreased from 108% to 29% while river discharge of Zhuqi increased from 0–500 m^3^·s^−1^ to 5000~ m^3^·s^−1^ in 2006; this is because the average width of the South Channel is wider than the North Channel, but the average bottom elevation of the North Channel is 2–3 m deeper than the South Channel, which makes SRNC bigger during the drought period than during the flood period.

In addition, SRNC had been decreased gradually in recent years, but with some different patterns between different grades of discharge. After the Shuikou Reservoir was built, river transport of sediments reduced drastically. The South Channel could not keep the balance between sediment transport and sand excavation continuously. What was worse, statistics of Fuzhou port showed the export of sand was soared from 1.75–13.0 million tons per year. So, SRNC under different grades of discharge, became smaller from 2005–2012, decreasing sharply between 2007–2008, which was because sediment dredging had been done repeatedly to ensure that the South Channel can reach the standards of navigation channel [31].

#### 4.1.2. Seasonal Trends of Severe DO Depletion

**Figure 3:**
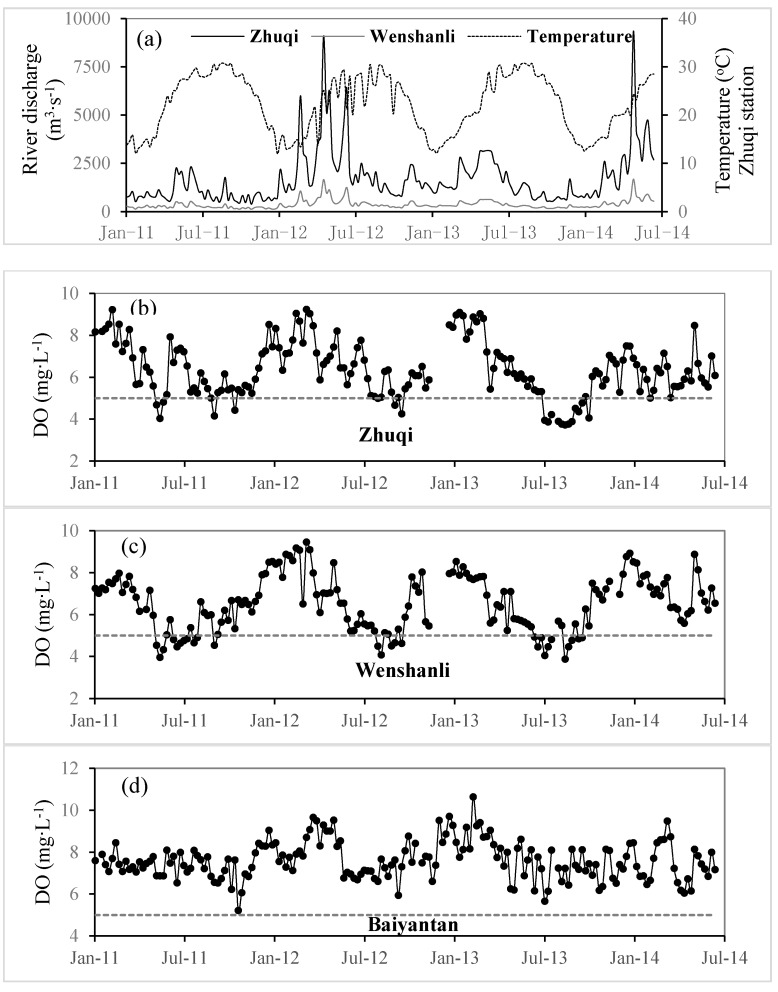
Evolution of weekly average values in river discharges (m^3^·s^−1^) of mainstream (black) and the North Channel (grey), water temperature at the Baiyantan station (°C—dotted line), dissolved oxygen (mg·L^−1^—black circles) at the three stations of the Minjiang River Estuary from January 2011–July 2014. Water temperature is plotted for the Baiyantan station only, as this parameter presents minor differences between the three stations.

Weekly average data throughout the four years of monitoring stations (Figure 3) revealed reproducible seasonal patterns induced by the hydrology, the changes in temperature and DO in the estuary. Comparative analysis of spring data (March–May), summer data (June–August), autumn data (September–November), and winter data (December–February) revealed different patterns between stations (Table 1). Throughout the studied period, 2011 was the driest year, with an annual average discharge of 930 m^3^·s^−1^; and 2012 was the wettest year with an annual average discharge of 2270 m^3^·s^−1^. Water temperature ranged between 10 °C and 32 °C and showed the same well-defined seasonal pattern at all stations. In contrast, DO contents and DO % of saturation presented contrasted signals, with significantly different values between stations and with seasonal variations more or less important depending on the location (Figure 3a; Table 1).

**Table 1:** Mean ± SD, minimum–maximum are presented using daily-averaged values for temperature, DO contents, DO % of saturation and river discharge depending on the spring (from March–May), the summer (from June–August), the autumn (from September–November) or the winter period (from December–February) for each station. Differences in the data between stations and seasons were tested with partial correlation analyses (comments in the text).

Hydrological Stations	Season	Dissolved Oxygen (DO) Contents (mg·L^−1^)	DO % of Saturation	Temperature (°C)	River Discharges (m^3^·s^−1^)
Zhuqi	Spring	6.65 ± 0.91	75.31 ± 9.28	22.48 ± 2.35	2508 ± 1180
		4.04–9.24	51.84–105.45	14.89–26.97	549–9352
		N = 295	N = 295	N = 295	N = 368
	Summer	5.80 ± 0.85	75.03 ± 10.36	29.23 ± 1.43	1968 ± 852
		3.73–7.93	50.73–103.35	26.62–30.36	480–6360
		N = 250	N = 250	N = 250	N = 305
	Autumn	5.36 ± 0.61	67.13 ± 6.43	26.25 ± 1.53	915 ± 322
		3.77–7.05	51.04–79.92	21.03–29.17	386–2419
		N = 215	N = 215	N = 215	N = 273
	Winter	7.42 ± 0.94	72.74 ± 9.08	14.45 ± 2.04	1184 ± 446
		5.01–9.23	50.03–90.19	11.94–18.73	520–5994
		N = 283	N = 283	N = 283	N = 330
Wenshanli (North Channel)	Spring	6.67 ± 0.89	75.6 ± 9.28	22.77 ± 3.65	514 ± 200
		3.96–9.46	50.82–105.17	15.24–27.49	176–1678
		N = 288	N = 288	N = 288	N = 368
	Summer	5.56 ± 0.62	72.05 ± 8.39	29.05 ± 0.92	420 ± 153
		3.88–7.27	52.8–95.07	27.86–30.77	200–1249
		N = 240	N = 240	N = 240	N = 305
	Autumn	6.07 ± 0.88	75.95 ± 9.27	26.46 ± 2.35	236 ± 62
		4.47–8.03	60.00–98.28	20.40–30.64	120–535
		N = 200	N = 200	N = 200	N = 273
	Winter	8.00 ± 0.51	78.14 ± 5.16	14.77 ± 1.45	277 ± 72
		6.92–9.18	64.22–91.48	12.17–18.63	126–1064
		N = 269	N = 269	N = 269	N = 330
Baiyantan	Spring	7.93 ± 0.85	90.25 ± 10.14	22.07 ± 3.97	
		6.05–9.66	69.12–113.89	12.60–29.10	
		N = 321	N = 321	N = 321	
	Summer	7.21 ± 0.42	93.59 ± 7.04	29.01 ± 1.05	
		5.66–8.12	73.79–110.3	26.00–32.1	
		N = 272	N = 272	N = 272	
	Autumn	7.23 ± 0.62	90.68 ± 7.63	26.34 ± 2.46	
		5.22–8.77	65.98–111.04	19.80–32.30	
		N = 295	N = 295	N = 315	
	Winter	7.91 ± 0.78	78.17 ± 8.67	15.37 ± 1.94	
		5.86–10.64	48.37–107.86	10.80–20.20	
		N = 304	N = 304	N = 304	

Concerning oxygen, there was a general trend same to that of river discharge and opposite to that of temperature, with the lowest DO in summer (high temperature) and autumn (high temperature and low discharge), but with some different patterns between stations (Figure 3). Estuarine waters at the Baiyantan station remained well oxygenated whatever the season, with weekly averaged values between 6 mg·L^−1^ and 11·mg·L^−1^ (saturation rates: 63%–114%; Figure 3d). In contrast, the two upstream stations, Zhuqi and Wenshanli, located in the fluvial estuary, weekly averaged DO concentrations varied widely between 3 mg·L^−1^ and 10 mg·L^−1^. DO concentrations were significantly higher in winter than in summer (Table 1) at both stations. This was primarily due to the temperature effect on oxygen solubility in water and the oxygen consumption due to degradation of the organic carbon and ammonium was lower in winter (December–February, temperature was below 20 °C). We also found that the DO values were higher in spring than in autumn at both stations when the temperature was the same, which was due to the dilution of more oxygenated upstream freshwaters. However, the averaged DO values of Zhuqi was lower in autumn than in summer which was different to Wenshanli station, whose DO was higher in autumn than in summer; this indicated that the prior role of temperature and river discharge effect on DO of Zhuqi and Wenshanli stations were different. Averaged DO in Zhuqi station was reduced from 5.80 mg·L^−1^ in summer to 5.36 mg·L^−1^ in autumn when the river discharge was dropped by 54% (from 1968–915 m^3^·s^−1^), even though the average temperature reduced from 29.23–26.25 °C; in Wenshanli station, averaged DO was increased from 5.56 mg·L^−1^ in summer to 6.07 mg·L^−1^ in autumn when the river discharge was dropped by 44% because SRNC became bigger in low discharge, which showed that the temperature effect on increasing DO at Wenshanli was more than the river discharge effect on decreasing DO. A large amount of monitoring data also showed that DO of Zhuqi and Wenshanli were substandard (below 5 mg·L^−1^) for 29.63% and 19.75%, respectively, with average river discharge of 975 m^3^·s^−1^ and 294 m^3^·s^−1^ respectively, and average temperature of 29.12 °C and 29.06 °C respectively in summer and autumn. It also turned out that DO of Zhuqi and Wenshanli were substandard (below 5 mg·L^−1^) for 44.40% and 54.55%, respectively when river discharge at both stations were less than 1000 m^3^·s^−1^ and 300 m^3^·s^−1^ respectively with temperature over 29 °C.

**Figure 4:**
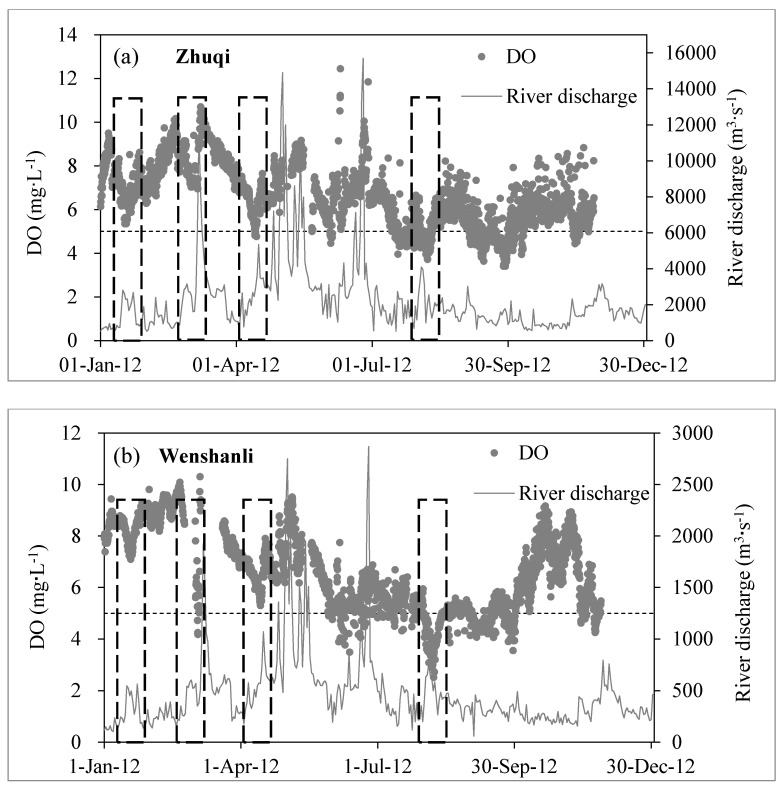
Evolution of daily average values in river discharges (m^3^·s^−1^—gray solid line) and dissolved oxygen (mg·L^−1^—gray points) at Zhiqi (**a**) and Wenshanli (**b**) stations of the Minjiang River Estuary during 2012. DO of Zhuqi and Wenshanli with four times sharp drops were marked by black dotted box.

The occurrence of hypoxia not always occurred in high temperature and low discharge. It also appeared during the floods after a long drought period, when a lot of wastewater and polluted initial rainwater overflows into the river. For example, it is most obvious that DO at three stations decreased significantly during the first flooding from the 1–15 May 2011 (the driest year) (Figure 3). Further evidence with high-frequency data in 2012 showed that DO of Zhuqi and Wenshanli had four times sharp drops in short-term during floods after one or two months of drought period (Figure 4). However, DO at both stations usually recovered rapidly because wastewater flowed into the ocean quickly. Water age [35,36] in Minjiang River Estuary, calculated by a model, was about six days when river discharge from Shuikou Dam was 2000 m^3^·s^−1^ (not yet published). It just took less than three days for domestic sewage and industrial wastewater of the municipal district of Fuzhou (2,984,900 inhabitants) to discharge into the sea under river discharge beyond 2000 m^3^·s^−1^. Therefore, low DO happened during floods still demanded concern.

#### 4.1.3. Correlation of DO with Flow/Temperature

In order to investigate the factors controlling water oxygenation spatially and seasonally in the Minjiang River, partial correlation tests were performed on daily-average dataset of DO, river discharge and temperature from 2011–2014 at three stations (Table 2). To separate the prior role of temperature or river discharge effect on DO, tests were performed on between daily-average dataset of DO and river discharge or temperature in the case of controlling the temperature or river discharge. All correlations were significantly at the 0.0001 probability level. Comparing data of three stations, some patterns are confirmed by the tests, as follow: (1) positive correlations were observed between DO and river discharge, while the DO of Zhuqi showed the stronger correlation with river discharge than the other two stations; this is because the variability of river discharge at Zhuqi was bigger than that at Wenshanli effected by SRNC. In addition, the water quality of Wenshanli was poorer than that of Zhuqi which might be polluted by sewage from Minhou town located between two stations. Water at Baiyantan was diluted by seawater with high DO at high tide, which resulted in DO of this section having a weak correlation with river discharge; (2) negative correlations were observed between DO and temperature. The correlation coefficient at Wenshanli was stronger than that at the Zhuqi, which confirmed the influence of temperature on DO at Wenshanli was greater than that at Zhuqi again. Also, the correlation coefficient at Baiyantan was just −0.33, much less than the other two stations, which also was due to diluted by seawater with high DO. The same is found in the Ems Estuary, where the field and model results also showed strong variations in DO contents, caused both by temperature and variation in the hydrologic cycle with low discharge during the summer period [5,9,13,37,38].

**Table 2:** Partial correlation coefficients between daily average DO (mg·L^−1^) and river discharge (m^3^·s^−1^) or temperature (°C) per station in the case of controlling the temperature or river discharge from 2010–2014. All correlations were significant at the 0.0001 probability level.

DO (mg/L)	Temperature	River Discharge
Zhuqi	−0.74	0.54
Wenshanli	−0.81	0.23
Baiyantan	−0.33	0.25

### 4.2. Location of DO Depletion

#### 4.2.1. The Change of DO along the Minjiang River

**Figure 5:**
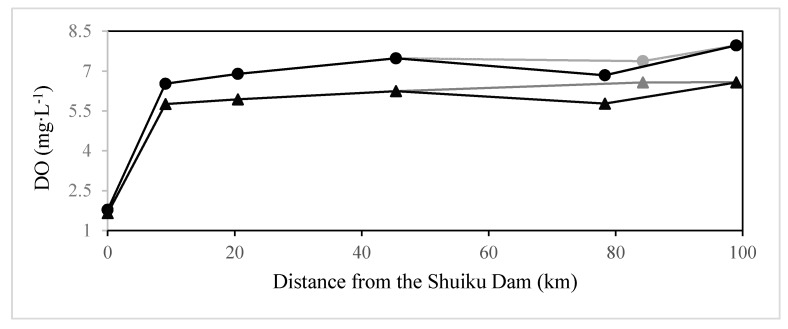
Observed average concentration of DO during the summer and the autumn period (June–November, triangle) and during the winter and the spring period (December–May, circle) along the estuary from 1995–2013. The North Channel (black line) and the South Channel (gray line) were plotted, respectively.

Contrary to weekly averaged data of three stations, water quality of seven routine monitoring points in the Minjiang River as shown in Figure 5 from 1995–2013, revealed the evolution of water quality along the Minjiang River. To separate the effect of temperature, average concentration of DO was analyzed during the high temperature seasons (June–November) and the low temperature seasons (December–May) along the estuary. The averaged DO at different monitoring points (except the Shuikou point) was 0.76–1.39 mg·L^−1^ higher in summer and autumn than in winter and spring. As the river discharge outlet was located at the bottom of Shuikou Dam, DO at Shuikou was low. However, the reaeration rate was far greater than oxygen consumption rate in the river because of the great momentum of river discharge and relatively low biochemical reaction due to its good water quality. As a result, averaged DO was increased to more than 5.5 mg·L^−1^ at Geyangkou, located 9.1 km from Shuikou Dam. DO would be further increased until the discharge flow running into the bifurcated channel of the Minjiang River. In the fluvial section of the North Channel around the municipal district of Fuzhou, DO was decreased significantly.

**Figure 6:**
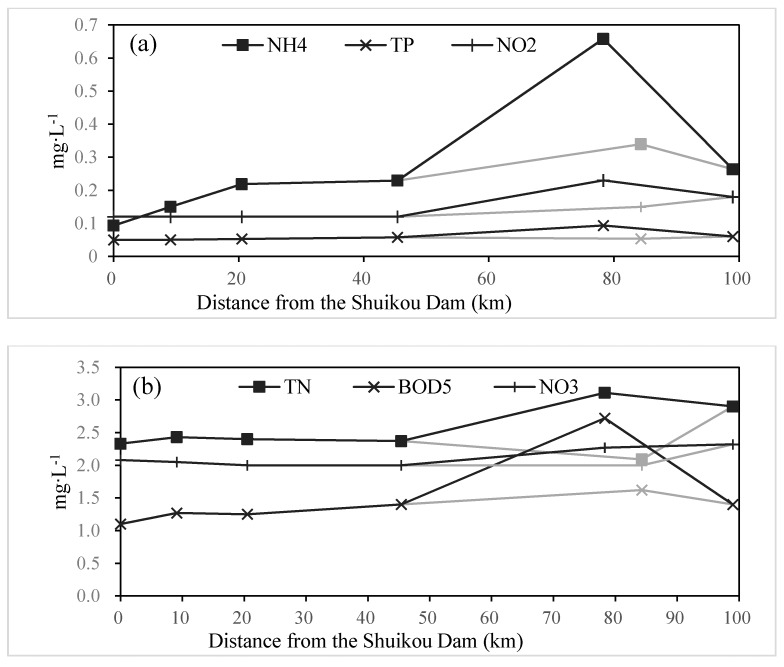
Observed average concentration of ammonium (**a**. mg·L^−1^—black square), total phosphorus (**a**. mg·L^−1^—furcation), nitrite (**a**. mg·L^−1^—crisscross), total nitrogen (**b**. mg·L^−1^—black square), BOD5 (**b**. mg·L^−1^—furcation) and nitrate (**b**. mg·L^−1^—crisscross) along the estuary from 1995–2013. The North Channel (black line) and the South Channel (gray line) were plotted, respectively.

The average concentration of ammonium, BOD_5_, and other water quality in the North Channel worsened at various degrees (Figure 6). Water quality in the South Channel changed a little, such as total nitrogen, nitrite and total phosphorus, while only BOD_5_ and ammonium were increased slightly. DO and other water qualities were improved at the Jingangtui monitoring point due to the dilution of seawater. 

This suggests an impact of the municipal district of Fuzhou (2,984,900 inhabitants), which releases 80% domestic and industrial pollutants of low reaches of the Minjiang River into the North Channel after treatments [32]. Daily organic carbon and ammonium fluxes from wastewater treatment plants and urban rivers (Guangming River and Baima River) are estimated at respectively 105 t·C per day and 12 t·N per day (unpublished data). However, average inputs of biodegradable organic carbon and ammonium from the upstream Minjiang River are respectively 32 t·C per day and 3.3 t·N per day. The impact of urban sewage loads on Minjiang oxygenation around the municipal district of Fuzhou is potentially higher than river inputs coming from upstream. During summer or autumn low river discharge and long residence time of water, urban inputs of highly biodegradable organic matter and ammonia fuel microbial respiration and constitute significant additional factors that contribute to hypoxia in this tidal reach of the Minjiang River (Figure 3, Figure 4, Figure 5 and Figure 6; Table 1).

#### 4.2.2. Occurrence of a DO Depleted Water Mass Centered on the Municipal District of Fuzhou

**Figure 7:**
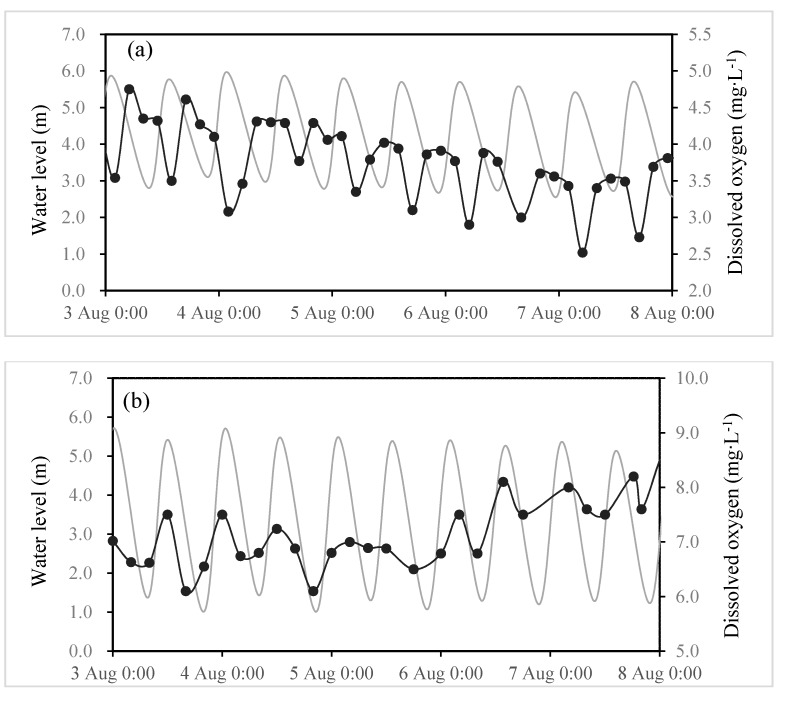
Temporal evolutions of the water level (mg·L^−1^—grey) and DO (mg·L^−1^—black) contents at Wenshanli (**a**) and Baiyantan (**b**) from 3–8 August 2012. Data frequency was three hours.

Further evidence of an urban impact on the Minjiang River in summer comes from DO variations throughout tidal cycles, which show different timing at Wenshanli and Baiyantan. Figure 7 shows, as an example, three hours raw data of DO concentration, the water level from the 1–8 August, 2012 at Webshanli and Baiyantan. Discharge of the Wenshanli was relatively constant between 499 and 651 m^3^·s^−1^ during this period, and the lowest DO concentration recorded was 2.52 mg·L^−1^ in Wenshanli. The water level varied from 2.20–5.97 m at Wenshanli and from 0.89–5.72 m at Baiyantan. The temperature was 27.7 °C–31.8 °C at both stations (not shown), without significant difference between stations.

At Wenshanli, the highest DO concentration occurred at low tide slack water and the lowest DO occurred at high tide (Figure 7a). At Baiyantan, the trend in DO concentration was the opposite, with minima recorded at low tide slack water and maxima at high tide (Figure 7b). In addition, the high tide DO concentration at Wenshanli exactly matched the low tide DO concentration at Baiyantan. This phase offset in DO between Wenshanli and Baiyantan is another indication of the impact of the municipal district of Fuzhou: at Wenshanli DO is minimum at high tide when water from Fuzhou is advected, but maximum at low tide when dilution with fresh riverine water occurs; DO at Baiyantan is highest at high tide when dilution with estuarine saltier water occurs, which illustrates the upstream migration of oxygen-depleted water from the municipal district of Fuzhou to Baiyantan with the flood tide. One could expect an equivalent migration of the municipal district of Fuzhou (2,984,900 inhabitants) impacted water downstream with the ebb tide. Lanoux and Gilbert *et al.* had found the same phenomenon in the Gironde Estuary [5,11,39,40,41]. The study indicates a potential negative impact of the wastewater effluent on the receiving river environment and suggests a serious public health issue for people who use receiving water sources for drinking and other purposes.

### 4.3. The Trends

**Figure 8:**
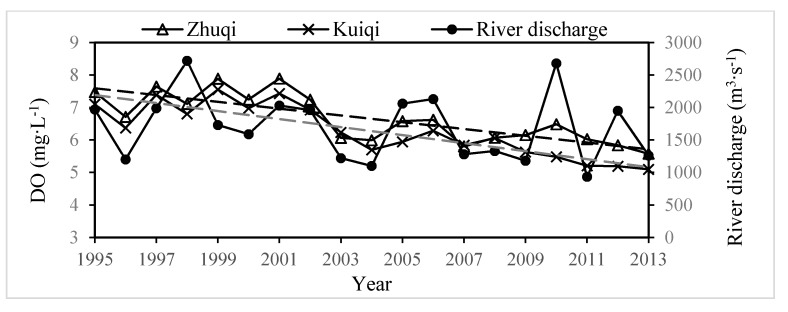
Trend of yearly average DO (mg·L^−1^) at Zhuqi (triangle) and Kuiqi (furcation) during the summer and the autumn period (June–November), river discharge of mainstream in the Minjiang River (m^3^·s^−1^—circle) from 1995–2013. The trend lines of Zhuqi (black dotted line) and Kuiqi (gray dotted line) were plotted.

Due to the population increase and economic development in Fuzhou City, the Minjiang River Estuary has a high load of anthropogenic nutrients from increased agricultural activities and wastewater [28]. Yearly average DO at Zhuqi and Kuiqi during the high temperature season (June–November) from 1995–2013 (Figure 8) tends to decrease. As an example, average DO at two stations in 1996 are 6.71 and 6.37 mg·L^−1^ respectively, lower than that in 2009, whose DO are 6.15 and 5.63 mg·L^−1^, respectively, even though river discharges in the two years are the same (about 1200 m3·s^−1^). Figure 8 also shows that river discharge has some helpful effect on DO by renewing water in the hypoxia section with more oxygenated upstream freshwater. At very low discharges, residence time of water bodies is longer, water renewal is lower and the environmental capacity of nutrients in water reduces [42]. Oxygen consumption increases due to degradation of the organic carbon and ammonium by continuous input from Guangming River and Baima River. The range or duration of hypoxia in the Minjiang River Estuary will become bigger or longer in the future, which will result in deaths of phytoplankton and zooplankton, variation and loss of the habitats and deterioration of water quality of water source areas (Yuancuo, Aofengzhou and Mawei), as well as endangering the public health of the people who depend on this all important water resource for drinking and other purposes [14,15,43]. In order to avoid drastic oxygen depletion in the Minjiang River around the municipal district of Fuzhou, reduction of pollutants of local urban and minimum discharge are very critical. During the high temperature and the drought period, the limit of 700 m^3^·s^−1^ is the criterion for management of dams to release water from Shuikou Dam on the watershed in order to sustain flow discharges, and sediment dredging in the South Channel must be forbidden to guarantee the split ratio of the North Channel (SRNC) beyond 30%.

## 5. Conclusions

The purpose of this study was to analyze the synthesis of different factors influencing hypoxia in the Minjiang River Estuary. The long sequence and high-frequency of measurements of stations in the Minjiang River provided detailed information on hydrological and water quality that would not be obtained by classical sampling. Weekly average data of monitoring revealed seasonal patterns whereas water quality of seven routine monitoring points revealed an evolution of DO along the Minjiang River. Spatio-temporal variations in DO are in accordance with the location of the considered station in the Minjiang River Estuary. Baiyantan, the station in the central estuary, records the highest oxygen levels throughout the year (Table 1) due to inputs of oxygenated oceanic waters. Zhuqi and Wenshanli in the upstream show the same trends, with an oxygen decline during the summer and the autumn period, when temperatures are high and river discharges are low. In addition, low DO might experience a sharp drop in the short-term during floods after one or two months of drought when lots of wastewater and polluted initial rainwater overflow into the river, but DO could recover rapidly because wastewater flows into the ocean quickly. Affected by the split ratio of the North Channel, the river discharge effect on DO at Zhuqi was more obvious than that at Wenshanli and the temperature effect on DO at Wenshanli was greater than that at Zhuqi. The Kuiqi routine monitoring point showed significantly lower DO levels than the others, which demonstrated that water oxygenation at the North Channel was affected by local urban inputs. The main factors that influence DO contents are the temperature, river discharge and sewage loads. DO in Wenshanli was out of phase compared to Baiyantan, due to the tidal upstream advection of oxygen-depleted waters within a tidal cycle.

Water source areas in the North Channel are threatened due to hypoxia leading to deterioration of water quality and variation and loss of the habitats. There is an urgency for the local city to properly address the issue of sewage treatment and wastewater discharge to safeguard its water source areas, coastal environment and public health. As hypoxic situations are observed and tend to be more severe during marked summer and low-river flow on the North Channel section or during floods after one or two months of drought, pollutants of the municipal district of Fuzhou must be reduced. Finally, minimal mainstream flow above 700 m^3^·s^−1^ from Shuikou Dam and the split ratio of the North Channel (SRNC) beyond 30% allows avoidance of the drastic hypoxic situation by renewing water masses in the municipal district of Fuzhou.
